# Development and validation of an LC-MS/MS method for quantification of favipiravir in human plasma

**DOI:** 10.1016/j.jpba.2023.115436

**Published:** 2023-05-02

**Authors:** Elizabeth Challenger, Sujan Dilly Penchala, Colin Hale, Richard Fitzgerald, Lauren Walker, Helen Reynolds, Justin Chiong, Tom Fletcher, Saye Khoo, Laura Else

**Affiliations:** a Department of Molecular and Clinical Pharmacology, University of Liverpool, Liverpool L7 8TX, UK; b Liverpool University Hospitals NHS Foundation Trust, Prescot Street, Liverpool L7 8XP, UK; c Liverpool School of Tropical Medicine, Pembroke Place, Liverpool L3 5QA, UK

**Keywords:** Favipiravir, Pharmacokinetics, Bioanalysis, COVID-19, SARS-CoV-2

## Abstract

Favipiravir (FVP) is a broad-spectrum antiviral that selectively inhibits viral RNA-dependent RNA polymerase, first trialled for the treatment of influenza infection. It has been shown to be effective against a number of RNA virus families including arenaviruses, flaviviruses and enteroviruses. Most recently, FVP has been investigated as a potential therapeutic for severe acute respiratory syndrome coronavirus 2 infection. A liquid chromatography tandem mass spectrometry method for the quantification of FVP in human plasma has been developed and validated for use in clinical trials investigating favipiravir as treatment for coronavirus disease-2019. Samples were extracted by protein precipitation using acetonitrile, using ^13^C, ^15^N- Favipiravir as internal standard. Elution was performed on a Synergi Polar-RP 150 × 2.1 mm 4 μm column using a gradient mobile phase programme consisting of 0.2% formic acid in water and 0.2% formic acid in methanol. The assay was validated over the range 500–50,000 ng/mL; this method was found to be precise and accurate and recovery of FVP from the matrix was high. Stability experiments confirmed and expanded on the known stability of FVP, including under heat treatment and for a period of 10 months at − 80 °C.

## 1 Introduction

Favipiravir (FVP) is a pyrazine carboxamide derivative initially investigated for the treatment of influenza infection. It has since been demonstrated to have activity against the RNA-dependent RNA polymerase (RdRp) of multiple RNA virus families as well as against neuraminidase-inhibitor resistant strains of influenza [1]. The RdRp of RNA viruses is generally well conserved, making FVP an ideal drug for use against viruses with RNA genomes. In 2014, FVP was approved for use against new and re-emergent strains of pandemic influenza in Japan [2]. FVP is a pro-drug that undergoes intracellular phosphorylation and ribosylation to the active favipiravir-ribofuranosyl-5’-triphosphate (FVP-RTP), which incorporates into the nascent viral RNA leading to chain termination and replication inhibition [3].

In late 2019, a novel betacoronavirus (SARS-CoV-2), causing viral pneumonia emerged in Wuhan, China. The rapid spread of the virus in the absence of any natural population immunity rapidly led to high numbers of infection globally, and prompted the search for effective therapeutics, with favipiravir quickly materialising as a promising candidate due to its known potential against RNA viruses [4]. Various *in vitro* studies generated mixed results regarding the activity of FVP against SARS-CoV-2 infection in Vero E6 cells with reports ranging from an EC_50_ of 61.66 μM to no apparent *in vitro* activity [5–7].

Despite this, human trials conducted in China at the beginning of the pandemic suggested that FVP could lead to shorter viral clearance time and improvements in computed tomography (CT) chest imaging in patients with mild to moderate COVID-19. However, such trials involved small patient numbers, and were not randomised, blinded or placebo controlled. Further randomised, placebo controlled clinical studies have reported lack of efficacy, especially when administered in early symptomatic COVID-19 infection [8,9]. A number of these trials investigated a loading dose of FVP 1800 mg twice daily given on day 1, followed by 800 mg twice daily for a minimum of 5 but up to 10 days. In all cases, no difference was observed between placebo groups and FVP groups in viral clearance, disease progression or resolution of symptoms [8–11].

It is still postulated that FVP has possible clinical use for patients with severe COVID-19 illness, but dose and duration of treatment need to be investigated in more depth. It is possible that the oral doses investigated are too low to achieve appropriate concentrations of the active intracellular metabolite, FVP-RTP; a regimen of 1600 mg BD on day 1 followed by 1200 mg BD has been posited using physiologically-based pharmacokinetic (PBPK) modelling and simulation [12].

Teratogenicity is also a major concern, with a number of animal species exposed to typical FVP doses having internal, external or skeletal anomalies [13]. However, emerging data on a limited number of pregnant patients exposed to FVP (n = 38) suggests that the teratogenic effect may not be as significant in human pregnancies, although more extensive studies are needed to confirm this [14,15].

In order to further investigate the clinical uses of FVP, alternative routes of administration and characterise exposure in different populations (such as pregnancy), simple and fast measurement of FVP in biological matrices is required. Validated liquid chromatography - tandem mass spectrometry (LC-MS/MS) methods for the quantification of FVP have been previously reported [16–18]. Here, we present a robust, simplified LC-MS/MS method with more extensive stability data than in current literature.

## 2 Materials and methods

### 2.1 Chemicals

FVP and ^13^C, ^15^N - FVP (98% purity) were acquired from Toronto Research Chemicals (TRC, Ontario, Canada) and AlsaChim (AlsaChim, Illkirch, France), respectively. Ultra purified water (18 Ω) was obtained from an Avidity Duo system (Avidity Science, Buckinghamshire, UK). LC-MS grade methanol and acetonitrile were procured from Fisher Scientific (Loughborough, UK). Drug-free human plasma from healthy volunteers was sourced from the NHS Blood and Transplant Service (Liverpool, UK) with ethical approval from the NHS Health Research Authority.

### 2.2 Equipment

An Agilent 1260 Infinity series (LC pump, autosampler and column oven; Agilent Technologies, Santa Clara, USA) served as the HPLC system. The mass spectrometer was an AB Sciex 6500 triple quadrupole system (AB Sciex, Macclesfield, UK) interfaced with a heated-electrospray ionization (ESI) source. A reverse-phase Synergi Polar-RP 150 × 2.1 mm 4 μm (Phenomenex, Macclesfield, UK) column with oven temperature set at 40 °C was used for compound elution. Data acquisition was performed using Analyst (v1.6.1) and processed with MultiQuant (v3.0.3) (AB Sciex, Macclesfield, UK).

### 2.3 Stock solution preparation

FVP stock powder was weighed on a calibrated analytical balance (Sartorius, Göttingen, Germany) and diluted with methanol to achieve a concentration of 5 mg/mL. Intermediate solutions were prepared by further dilution of the stock solution in methanol to generate solutions of 2500, 500 and 62.5 μg/mL, respectively.

Plasma calibrators were prepared by spiking 20 μL of these intermediate stock solutions into 1 mL (2% spiking) blank human plasma to generate three calibrator levels at concentrations of 1250, 10000 and 50,000 ng/mL FVP. The spiked working calibration standards were diluted with blank plasma to generate final concentrations in plasma of 500, 1250, 2500, 5000, 10000, 25000, 40000 and 50,000 ng/mL of FVP. Low (LQC), medium (MQC) and high (HQC) quality control samples were prepared at 1401, 11205 and 41,500 ng/mL, respectively. A lower limit of quantification (LLQ) QC sample was also prepared at 500 ng/mL.

Internal standard (^13^C, ^15^N-FVP) stock solution was prepared at a concentration of 1 mg/mL using methanol as the diluent. Working internal standard was prepared by diluting the stock 40-fold in methanol-water (50:50, v/v) to a final concentration of 25 μg/mL.

### 2.4 Sample preparation

100 μL of calibrators, QCs and blank plasma samples were aliquoted into 5 mL borosilicate test tubes followed by 20 μL of internal standard solution (^13^C, ^15^N-FVP, 25 μg/mL). Proteins were precipitated from the plasma by addition of 400 μL of acetonitrile to all tubes. Samples were briefly mixed by vortexing before centrifugation for 10 min at 2688 x *g* and transfer of 50 μL of the supernatant to clean 5 mL tubes. 950 μL of methanol-water (80:20, v/v) was added to all tubes before aliquoting 100 μL from each into glass autosampler vials. Samples were loaded into the autosampler and injected (5 μL) into the LC-MS/MS.

### 2.5 LC−MS/MS procedure

A Phenomenex Synergi Polar-RP C18 column (4 μm; 150 × 2.1 mm) was used to achieve separation using 0.2% formic acid in water (mobile phase A) and in methanol (mobile phase B). A gradient method with flow rate set to 600 μL/min was utilised as follows: initial conditions of 20% mobile phase B held for 0.8 min then increased to 80% mobile phase B for 2 min, followed by column conditioning with starting conditions for 3.2 min to achieve a total run time of 6.0 min.

Positive ion mode using multiple reaction monitoring (MRM) was used for detection of FVP. The *m/z* transitions used were 158.0→141.0 for FVP and 160.1→142.0 and 160.1→113.0 for ^13^C, ^15^N-FVP. Mass spectrometer conditions were optimised as summarised in Table 1.

## 3 Validation methodology

This method was validated in accordance with FDA/EMA bio-analytical method guidelines [19,20].

### 3.1 Selectivity

Selectivity experiments were conducted by screening six separate lots of blank human plasma and assessing them for endogenous interferences that elute at the same retention time as the analyte and internal standard. Any background interference at the retention time of FVP was deemed acceptable if ≤ 20% of the mean FVP response in the LLQ sample. For the internal standard, any background noise was considered acceptable if < 5% of the mean internal standard response in LLQ samples (n = 6).

### 3.2 Recovery and matrix effects

Six sets of aqueous LQC, MQC and HQC samples were prepared in the final reconstitution solution of methanol-water (80:20, v/v), a further six were prepared by spiking of drug into extracted blank plasma. Overall recovery was calculated by comparing the peak area response of the non-extracted samples to plasma samples spiked with FVP prior to extraction. Matrix effect was established by comparing the absolute peak areas of FVP spiked into final reconstitution solution to that of FVP spiked into blank plasma extract. Both overall recovery and matrix effect were quantitated using methods described by Matuszewski [21].

### 3.3 Accuracy and precision

Inter and intra-day accuracy and precision were evaluated by analysing calibration curves containing six replicates of LQC, MQC and HQC samples run over the course of 7 days. The calibration range was set according to reported C_max_ values achieved with the standard FVP oral dosing regimen (600–800 mg twice daily) and potential dosages postulated using PBPK modelling [8–12].

### 3.4 Carryover

Carryover was measured using extracted blank plasma samples injected following the ULQ sample (50,000 ng/mL) and the response compared to that of the LLQ sample, and expressed as a percentage. The limits of ≤ 20% of the LLQ response and < 5% of the IS response were used for carryover experiments [19].

### 3.5 Stability

Stability of FVP in plasma was evaluated under different conditions using sets of six LQC and HQC samples. Heat stability was tested by incubation of the samples at 58 °C for 40 min, simulating viral inactivation procedures. Freeze-thaw stability was tested by removing frozen samples and thawing at room temperature and re-freezing at − 80 °C for three freeze-thaw cycles. Ambient stability was determined by leaving plasma QC samples on the benchtop and quantifying against a freshly prepared calibration curve. The stability of FVP in the autosampler was established by storing QC samples in the autosampler for 72 h and analysing them on a freshly prepared calibration curve. An accepted precision and accuracy batch was re-injected after being left in the autosampler at 15 °C for 24 h, to assess reinjection reproducibility. An extracted set of QC samples was refrigerated for 24 h before being injected alongside calibrators prepared at the time of analysis, to examine the stability of FVP in processed samples. Finally, the long-term stability of FVP was assessed by analysing quality control samples that were previously spiked using a freshly spiked calibration curve.

### 3.6 Dilution integrity

Plasma was spiked with FVP at 80,000 ng/mL (1.6-fold higher than the ULQ) and subsequently diluted 1:2 and 1:4 with blank plasma. These samples were analysed against the calibration curve, with the applicable dilution factor, to determine the calculated concentration compared to the anticipated nominal concentration.

### 3.7 Clinical application

This method was developed specifically for use in the ongoing Accelerating COVID-19 Drug Development – Phase I/II trial platform (AGILE) CST-6 trial - a randomized, multicentre, seamless, adaptive, phase I/II platform study to determine the phase II dose and to evaluate the safety and efficacy of IV intravenous favipiravir for the treatment of COVID-19 [EudraCT 2020–001860–27]. Hospitalised patients with polymerase chain reaction (PCR)-confirmed SARS-CoV-2 infection who had severe disease (requiring oxygen by mask or non-invasive or high flow oxygen) were enrolled within 14 days of symptom onset and were randomised (2:1) to receive either FVP or the standard of care (SoC). Multiple doses of FVP were administered by intravenous (IV) infusion over 1 h, every 12 h for 7 days duration. FVP dosing was increased in cycles at a starting dose of 600 mg twice daily, with dose de-escalation or escalation guided by emerging safety data and a decision by the Safety Review Committee. Intensive pharmacokinetic sampling was undertaken on day 1, day 3 and day 5 after starting treatment, with blood (K_2_EDTA) drawn at pre-dose, and up to 12 h following the first IV infusion. The whole blood was centrifuged and the resultant plasma immediately frozen at − 80 °C until LC-MS analysis.

The pharmacokinetic (endpoint) data are to be presented in a separate AGILE manuscript; however, a sub-set of the preliminary data are presented here in order to support the clinical utility of the analytical method.

## 4 Method validation

### 4.1 LC-MS conditions

**Fig. 1:**
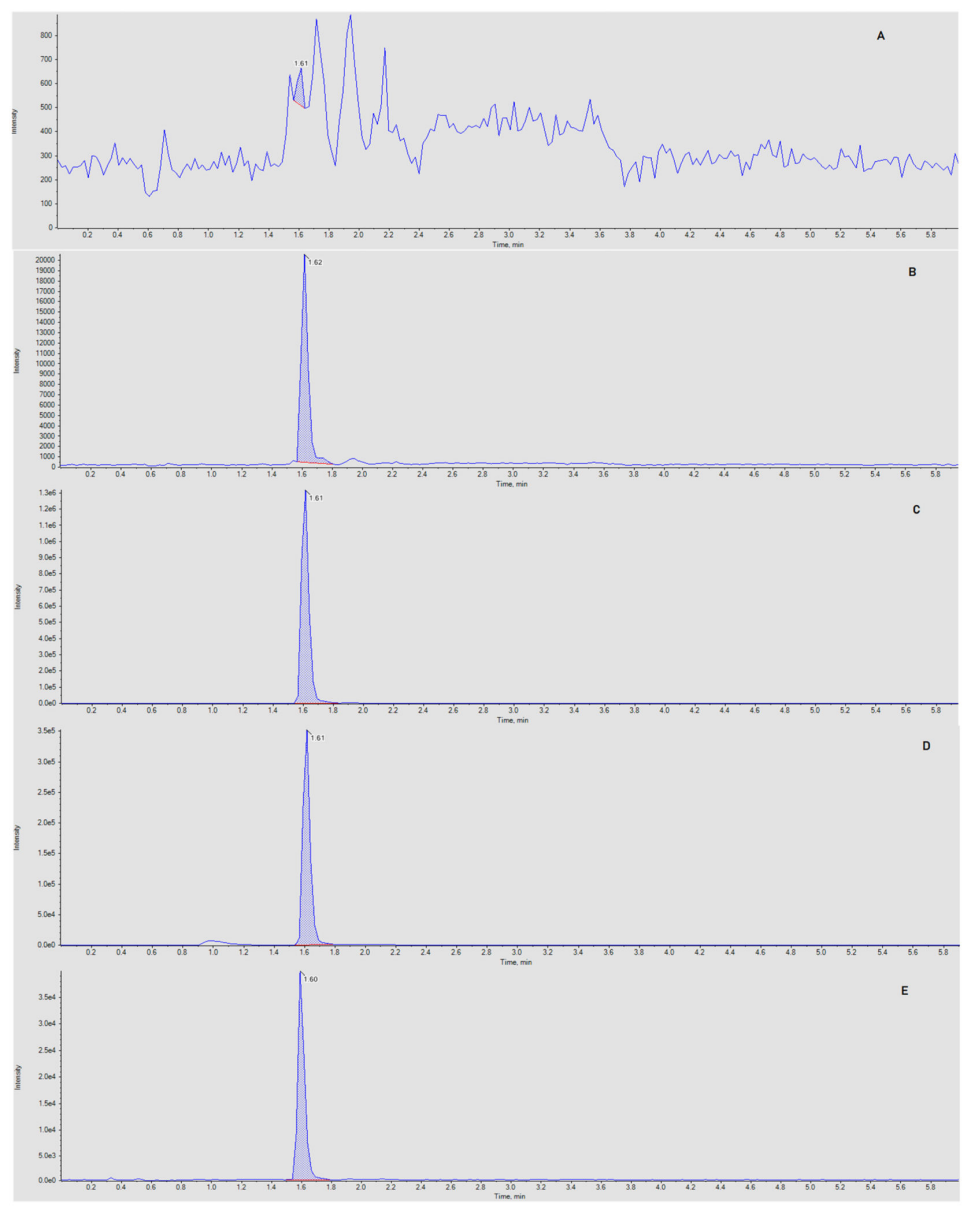
Representative chromatograms of favipiravir from an extracted blank sample (a), the lower limit of quantitation (b), a high quality control sample (c), a clinical sample (16,689 ng/mL) (d) and internal standard (e).

FVP and ^13^C, ^15^N-FVP eluted at 1.61 and 1.60 min, respectively at a mobile phase B concentration of 80%. Representative chromatograms for blank, IS, LLQ, HQC and a clinical sample (16,689 ng/mL) are shown in Fig. 1. Formic acid was used as an additive to the mobile phases in order to improve peak shape and provide protons to assist production of [M+H]^+^ ions.

### 4.2 Selectivity

Background interference was negligible in all six plasma batches analysed for both FVP and internal standard.

### 4.3 Recovery and matrix effects

Recovery, matrix effect and process efficiency were evaluated across three concentrations (LQC, MQC, HQC). The results are summarised in Table 2. The matrix effect was minimal and the overall recovery was > 85% for all concentrations tested.

### 4.4 Accuracy and precision

Inter- and intra-assay accuracy and precision was within ± 15% of the nominal concentrations for LQC, MQC and HQC samples and ± 20% for the assay LLQ (Table 3). A regression equation with quadratic weighting (1/x^2^) was applied to produce the best fit for the concentration-detector response. The average correlation coefficient (r^2^) was 0.99936 (n = 4).

### 4.5 Carryover

Carry over was < 10% and < 6% of the LLQ after injection of the first and second extracted blank following the ULQ, respectively. Thus, there was no requirement to include additional extracted blank samples between clinical samples in the assay sequence.

### 4.6 Stability

The results of stability experiments are summarised in Table 4. FVP spiked into plasma was shown to be stable when kept at ambient temperature for 22 h, and was also stable after four cycles of incubation at 58 °C for 40 min with re-freezing between each cycle. Plasma samples prepared and stored at - 80 °C were stable for up to a period of 10 months, with percent stability calculated at 87%. In extracted samples, FVP was stable when stored in the fridge for 24 h, the autosampler at 15 °C for 72 h and following reinjection of an accepted assay after 24 h in the autosampler.

### 4.7 Dilution integrity

Diluted samples (2- and 4-fold) demonstrated calculated concentrations within ± 15% of the nominal value and > 67% of dilution integrity QCs met this criteria. The accuracy, calculated as percentage bias, was 10% with a coefficient of variation of < 5%. Results of dilution integrity experiment are shown in Table 5.

### 4.8 Clinical application

A chromatogram from an extracted plasma from a patient receiving IV FVP (600 mg twice daily; Day 3) is shown in Fig. 1d. Of four patients that received the lowest dose of IV FVP on day 3 of the trial (a total of 16 samples), FVP plasma concentrations ranged between 1335 and 19,418 ng/mL. All pre-dose concentrations (0 h; n = 4) and 1 of 4 samples collected at 6–12 h post-infusion were below the assay LLQ; all other samples were quantifiable. These data are purely descriptive; the complete pharmacokinetic data set is to be published in a separate clinical publication for the CST-6 AGILE trial.

## 5 Discussion

A robust, accurate and selective LC-MS/MS method has been developed and validated for the measurement of FVP in human plasma. The described assay offers several improvements with respect to previously published bioanalytical methods [17,22,23]. Our method requires a lower sample volume (100 μL) and uses a stable isotopically labelled (SIL) internal standard. SIL compounds have virtually identical physiochemical properties to their unlabelled counterparts, allowing them to compensate for any variability in sample processing and analyte detection, resulting in improved precision and accuracy.

The stability data provided in this report confirms and expands on published figures. Our experiments confirmed stability of FVP when left on the benchtop, and throughout multiple freeze-thaw cycles. Auto-sampler stability data published by Morsy [17] of 27 h is enhanced by our finding that FVP is stable for 72 h in an autosampler set at 15 °C. Furthermore, this study has demonstrated that FVP remains stable in plasma when stored at - 80 °C for a period of 10 months.

Our study has characterised the stability of favipiravir in plasma when subjected to heat inactivation procedures. In the United Kingdom, SARS-CoV-2 is classified as a hazard group 3 pathogen [24]. Heat inactivation is therefore useful to enable safe working conditions and processing of samples in containment level two laboratories, which are more conventional than containment level three facilities. Inactivation of SARS-CoV-2 has been reported by heat treatment at 56 °C for 40 min [25], however this study used 58 °C for 40 min to reflect already established inactivation procedures for human immunodeficiency virus (HIV). Our data demonstrates that FVP is stable after four cycles of heat treatment, with re-freezing between each heat treatment. Although one round of heat treatment is sufficient for inactivation of SARS-CoV-2, multiple rounds may be useful if investigating favipiravir for treatment of other infectious diseases, for example Ebola Virus Disease. This also avoids the use of strong detergents that can contaminate mass spectrometers, as well as higher temperatures that may begin to influence stability of the drug.

This method was successfully developed, validated and utilised to quantify FVP in clinical samples obtained as part of the AGILE clinical trial platform (NCT04746183). The CST-6 trial aims to further our understanding of FVP pharmacokinetics in patients with severe COVID-19 when administered IV FVP. Further developments are in progress to quantify FVP in more complex clinical matrices, including nasal and ocular fluids that are relevant anatomical sites of onward transmission of the SARS-CoV-2 virus. Alongside this, relevant work is ongoing to establish a method for the quantification of the active intracellular metabolite, FVP ribofuranosyl-5’-triphosphate from isolated mononuclear cells.

## Figures and Tables

**Table 1:** Summary of mass spectrometer conditions.

Parameter	FVP	^13^C,^15^N-FVP Quantifier	^13^C,^15^N-FVP Qualifier
Mass transition (Da)	158.0→141.0	160.1→142.0	160.1→113.0
Collision Energy (V)	25	30	35
Spray Voltage (ISV)	5500	5500	5500
Vaporizer Temperature (TEM°C)	450	450	450
Ion Source Gas 1 (GS1)	50	50	50
Ion Source Gas 2 (GS2)	40	40	40
Collision Gas (CAD)	8	8	8
Curtain Gas (CUR)	25	25	25

**Table 2:** Recovery and matrix effect of FVP in human plasma. Recovery and matrix calculations: A: Peak area of aqueous mobile phase solutions without matrix and without extraction; Analysis RE: Analysis recovery (internal standard [IS]-normalized) ratio of the mean peak area ratio of the analyte spiked prior to extraction (C2) to the mean peak area ratio of the analyte spiked after extraction (B2) × 100; B: Peak area of analyte spiked after extraction; B2: Ratio of the peak area of analyte and IS spiked after extraction; C: Peak area of analyte spiked prior to extraction; C2: Ratio of the peak area of analyte and IS spiked prior to extraction; Ext RE: Extraction yield calculated as the ratio of the mean peak area of the analyte spiked prior to extraction (C) to the mean peak area of the analyte spiked after extraction (B) × 100; ME: Matrix effect expressed as the ratio of the mean peak area of the analyte spiked after extraction (B) to the mean peak area of an equivalent concentration of analyte in mobile phase (A) × 100; PE: Process efficiency expressed as the ratio of the mean peak area of the analyte spiked prior to extraction (C) to the mean peak area of the same analyte standard (A) × 100.

Nominal QC Concentration (ng/mL)	Mean peak area			Mean peak area ratio		ME (%) B/A	Ext RE (%) C/B	PE (%) C/A	Analysis RE (%) C2/B2
	A	B	C	B2	C2				
1401	140,794	132,582	113,643	0.056	0.057	94.2	85.7	80.7	101.5
11,205	1056,367	1012,396	921,903	0.439	0.446	95.8	91.1	87.3	101.5
41,500	3972,388	3824,357	3217,613	1.718	1.580	96.3	84.1	81.0	92.0

**Table 3:** Inter- (n = 6) and intra-day (=1) precision and accuracy for favipiravir.

	LLQ (500 ng/mL)				LQC (1401 ng/mL)				MQC (11,205 ng/mL)				HQC (41,500 ng/mL)			
	Mean	SD	CV (%)	Bias (%)	Mean	SD	CV (%)	Bias (%)	Mean	SD	CV (%)	Bias (%)	Mean	SD	CV (%)	Bias (%)
Inter-day	522.43	26.17	5.01	3.23	1532.46	32.71	2.13	8.83	11,461.02	192.54	1.68	2.28	41,078.09	1625.33	3.96	-1.02
Intra-day	510.88	17.89	3.50	2.18	1532.70	38.95	2.54	9.40	11,604.90	222.30	1.92	3.57	41,762.15	1038.71	2.49	0.63

**Table 4:** Stability data for FVP in different environments.

Stability measured	Sample type	Sample	Precision (CV)	Accuracy (%)
Bench-top (22 h; ambient)	Plasma	LQC	4.58	5.71
		HQC	1.37	2.15
Heat inactivation (single cycle, 58 °C)	Plasma	LQC	3.07	12.4
		HQC	2.08	1.35
Heat inactivation (four cycles, 58 °C)	Plasma	LQC	3.70	4.54
		HQC	7.16	6.40
Freeze-thaw (3 cycles; –80 °C)	Plasma	LQC	1.22	0.17
		HQC	1.18	4.70
Autosampler (72 h; 15 °C)	Extracted sample	LQC	3.13	11.20
		HQC	2.70	-6.94
Reinjection reproducibility (24 h; 15 °C)	Extracted sampled	LQC	0.37	7.86
		HQC	1.80	1.51
Processed stability (24 h; 2–8 ° C)	Extracted sample	LQC	1.64	5.60
		HQC	1.51	-0.39

**Table 5:** Results of dilution integrity experiment. Dil-INT QC - dilution integrity quality control sample spiked at 1.6-fold higher than the ULQ (50,000 ng/mL).

Dil-INT QC replicate	Nominal FVP Concentration (ng/mL)	2-fold dilution	4-fold dilution		
		Calculated FVP concentration (ng/mL)	% bias	Calculated FVP concentration (ng/mL)	% bias
1	80,000	88,775	10.97	83,461	4.33
2	80,000	87,166	8.96	90,025	12.53
3	80,000	85,181	6.48	90,955	13.69
4	80,000	85,484	6.85	94,166	17.71
5	80,000	87,729	9.66	88,560	10.70
6	80,000	86,957	8.70	88,746	10.93
Mean		86,882	8.60	89,319	11.65
S.D		1241		3212	
%CV		1.43		3.60	

## Data Availability

Data will be made available on request.
